# The Community's Role in Rural Youth Suicide Prevention: Perspectives From the Field

**DOI:** 10.1111/ajr.70024

**Published:** 2025-03-10

**Authors:** Laura Grattidge, Ha Hoang, Jonathan Mond, Denis Visentin, David Lees, Stuart Auckland

**Affiliations:** ^1^ Centre for Rural Health University of Tasmania Launceston Tasmania Australia; ^2^ Manna Institute Armidale New South Wales Australia; ^3^ School of Medicine Western Sydney University Penrith New South Wales Australia; ^4^ School of Medicine and Psychology The Australian National University Canberra Australian Capital Territory Australia; ^5^ School of Health Sciences University of Tasmania Launceston Tasmania Australia; ^6^ School of Nursing University of Tasmania Launceston Tasmania Australia

## Abstract

**Objective:**

This study explored how rural communities can be involved in suicide prevention efforts for young people aged 12–25. It provides a focus on who is best placed to drive these efforts and what support these people need to implement initiatives in their communities.

**Setting:**

The research was conducted across Australia, with a focus on rural areas, where suicide rates are higher due to unique challenges, including geographic isolation, stigma and limited access to health services. These areas require community‐driven solutions tailored to local contexts.

**Participants:**

Thirty‐seven participants aged 29–72 contributed insights, with diverse professional roles and lived experiences in rural youth suicide prevention, including service providers, programme leaders, researchers and policymakers.

**Design:**

A qualitative approach was used, with semi‐structured interviews and focus groups conducted between January and September 2021. Data were thematically analysed using a reflective approach to identify key factors supporting rural youth suicide prevention.

**Results:**

Two key themes emerged: (1) Program planning and implementation: highlighting the need for adaptable, stigma‐sensitive and culturally responsive approaches, and (2) Breaking down silos: emphasising collaboration between schools, families, health services and community leaders. Trusted local figures such as teachers, sports coaches and peers were identified as crucial for fostering engagement and early intervention. Lived experience voices were recognised as integral to co‐designing and sustaining community‐led efforts.

**Conclusion:**

Rural communities are central to youth suicide prevention. By leveraging local relationships, addressing stigma and fostering collaboration, communities can create supportive environments that save lives. Policy and practice must prioritise funding and resources for community‐led, culturally sensitive approaches.

**Summary**

- What is already known on this subject
  ○ Community‐based suicide prevention has been broadly discussed in the context of rural Australia, often characterised by universal, community‐wide and non‐clinical strategies designed to enhance awareness and build protective factors.
  ○ Limited research specifically examines the role of community efforts tailored to young people.
- What this paper adds
  ○ This study advances research on rural community‐based suicide prevention, focusing specifically on young people.
  ○ It highlights the various roles communities can play, leveraging key connections such as peers, schools and families, emphasising how programmes and communities can be supported, fostering ownership via grassroots partnerships and decision‐making structures.

## 1 Introduction

Suicide is driven by a complex interplay of risk factors that are compounded for young people, aged 12–25, living in rural and remote areas [1, 2]. The number of people dying by suicide increases with remoteness [3]. In these areas there are inaccessible health services, high‐risk occupations associated with farming, and social factors that impact on help‐seeking behaviours. These behavious include being socially isolated, the stigma arising from traditional values and toxic stoicism [4]. For young people in the critical development period of adolescence, suicide risk centres strongly on the quality of social and familial relationships. Risk is compunded by experiences of bullying, adverse childhood experiences and trauma, including intergenerational trauma, how identity is developed, including sexual and gender identity, experiences of unemployment and underemployment, and mental health disorders. All of these factors are intimately interconnected [5, 6].

In rural areas, broadly referred to as areas outside of major cities [7], where services and supports are lacking or inadequate, there is a need to address factors contributing to suicide by making the best use of the scarce resources available [4, 8]. The concept of community development emerges as an area that can accommodate suicide prevention efforts, with the community seen as a valuable resource to be leveraged, guiding bottom‐up, grassroots approaches [9, 10, 11]. At this community level, solutions require an understanding of the rural context and the complexities of factors present in rural areas, including those influencing health and wellbeing [3, 12].

In a previous study [13] the authors examined community‐based suicide prevention in the context of rural Australia. Study findings highlighted the characteristics of community‐based approaches as ‘utilising universal, community‐wide, non‐clinical approaches, which aim to increase awareness and protective factors within communities’ [13]. These factors include the social determinants of health, stigma and other risk factors that contribute to the likelihood of suicide [8]. For young people, and particularly for those living in rural areas, risks associated with suicide, and the types of prevention programmes needed, are not neccesarily the same as those needed for adults. Young people may be less likely to seek mental health support than adults. Whereas adults may be more likely to have co‐morbid medical and psychiatric conditions, young people may more likely to be impacted by social networks [14].

While the effectiveness of suicide prevention initiatives within rural communities has previously been explored [11], including specifically for young people [8], to the authors' knowledge, no studies have specifically explored the role of communities in preventing youth suicide in rural Australia. This study, therefore, specifically aimed to explore the role of communities in implementing suicide prevention for young people in rural areas, including supports people need to plan and implement programmes. This builds on this previous study [13] exploring community‐based suicide prevention, with a focus specifically on efforts for young people. The questions guiding this research therefore explore who is best placed to implement and be involved in suicide prevention efforts for young people in rural areas? And how can communities best be supported in their efforts?

## 2 Methods

### 2.1 Study Design

A qualitative research design was utilised, with data collection using semi‐structured interviews and focus groups, held between January and September 2021. Participants were experts working in the field, as used in the first study exploring community‐based suicide prevention more broadly [13]. Qualitative methods were selected as they are commonly used to assist with the understanding of complex factors contributing to suicide and its prevention [15, 16].

### 2.2 Developing Interview/Focus Group Topic Guide

Topic guide development was guided by Kallio et al. [17], with open‐ended questions aligning to the research questions addressing areas of interest and gaps identified in the research. Stages of topic guide development were led by the first author, with the research team providing input at each stage. The topic guide was reviewed by researchers and community members external to the team prior to data collection.

The semi‐structured, open‐ended questions allowed the interviewer the freedom to ask additional questions to explore points further, as needed, to respond to issues or questions raised by participants Questions allowed the researchers to explore participant meaning‐making processes further around the role of community and how they can be best supported in their efforts [18]. Questions were focused on the characteristics and strengths of rural communities to implement suicide prevention initiatives, who is best placed to take on this role, and the support or information communities need to undertake these efforts. A full list of questions is detailed in Supporting Information 1.

### 2.3 Participants

Participants were experts in the field of rural youth suicide prevention, self‐identified, including people who had worked in paid positions as community‐level service providers; community or national‐level suicide prevention programme providers; or involved in research or policy development. All had expertise or experience (knowledge or practical) in the area of rural youth suicide prevention.

#### 2.3.1 Inclusion and Exclusion Criteria

Study inclusion selection criteria were being over 18 years of age, English speaking, living in Australia, and having expertise in rural youth suicide prevention (with young people aged 12–25) (as previously defined). Participants could be based anywhere in Australia (i.e., either rural or urban)

As the study sought to explore perceptions of people with experience working in these professional programme and research roles in Australia, people under the age of 18, people without paid experience, and people not based in Australia were not eligible to participate.

#### 2.3.2 Recruitment

A purposive sampling method [19] was used to recruit participants based on the inclusion criteria. Contacts working in the area, authors of relevant journal articles, and people identified through Google searches relevant to experience working in suicide prevention in Australia, were sent direct email invitations. Snowball recruitment was additionally used, including through social media (LinkedIn), with participants asked to forward the invitation to their own contacts working in the area, asking them to contact the research team if they wished to participate, with the aim to reach a greater number of people at the community level/field of interest [20].

Sample size was guided by the concept of data sufficiency, reached when the collected data adequately supported the development of meaningful insights and explanations, ensuring no significant gaps remained in the analysis [21]. The iterative data collection and analysis process used [22] emphasises the importance of sufficiency over volume [23] prioritising data sufficiency, depth and relevance necessary to answer the research questions effectively.

### 2.4 Procedures and Data Collection

Participants were provided with a study information sheet prior to interviews and focus groups, and signed consent and a general participant characteristics form were required to be completed. The safety of participants was ensured by providing sufficient study information on what is required, and any perceived benefits or potential for distress, and avenues for obtaining further support. Participants were also made aware of the voluntary nature of their participation through the participant information sheet and instructions prior to the interview/focus group. Interviews and focus groups were primarily conducted online via Zoom, with only one interview conducted face to face (as COVID restrictions allowed). Sessions proceeded with a brief introduction to the study, followed by the researcher going through the semi‐structured question guide (Supporting Information 1).

### 2.5 Data Analysis

Interviews and focus groups were audio‐recorded using a transcriber or recorded via the online platform for Zoom sessions. Data were de‐identified, and participant characteristics summarised using descriptive statistics in IBM SPSS Statistics. Qualitative data from interviews and focus groups were transcribed verbatim and analysed using NVivo. A thematic analysis approach was utilised to examine the data, following the six‐phase framework outlined by Braun and Clarke [22]. This process involved familiarisation with the data, generating initial codes, searching for themes, reviewing themes, defining and naming themes and producing the report. The data were systematically reviewed to identify recurring patterns and salient themes, with an inductive approach adopted to ensure themes emerged directly from the data, while maintaining sensitivity to the broader research context [24]. The analysis was supported by a reflexive approach to coding and theme development, acknowledging the researcher's positionality and potential influences on the interpretive process [25]. This ensured the findings were grounded in the data while aligning with the study's focus on rural youth suicide prevention, with initial themes generated from the data without allocating responses into predetermined categories. Transcripts were coded separately by the first researcher (Author 1) and confirmed by a second researcher (Author 2). Themes were discussed with team members to reach a consensus.

### 2.6 Ethics and Reporting

Ethics approval was granted by the University of Tasmania Human Research Ethics Committee on 11th of February 2021 (Project ID 23582). Written, informed consent was obtained from each person prior to participating. The Consolidated Criteria for Reporting Qualitative Research (COREQ), a 32‐item checklist for interviews and focus groups [26], was followed (Supporting Information 2) and the Aboriginal and Torres Strait Islander.

Quality Appraisal Tool reported on (Supporting Information 3).

### 2.7 Researchers' Reflexivity

This study was shaped by the positionality of the research team, comprising individuals with professional expertise in suicide prevention, mental health and rural health systems, as well as lived/living experience of suicide. The lead authors lived/living experience and prior work in rural communities informed an appreciation for the unique challenges faced by these populations, including limited services and the critical role of local relationships. To counter potential biases, the team prioritised inclusion of lived/living experience representatives and sought ongoing feedback from rural stakeholders. Recognising a predominantly urban‐based background, researchers adopted a reflexive approach, capturing participants' voices through open‐ended qualitative methods and iterative analysis. This commitment aimed to ensure the authenticity and cultural relevance of the findings while contributing actionable insights for rural youth suicide prevention.

## 3 Results and Discussion

**FIGURE 1:**
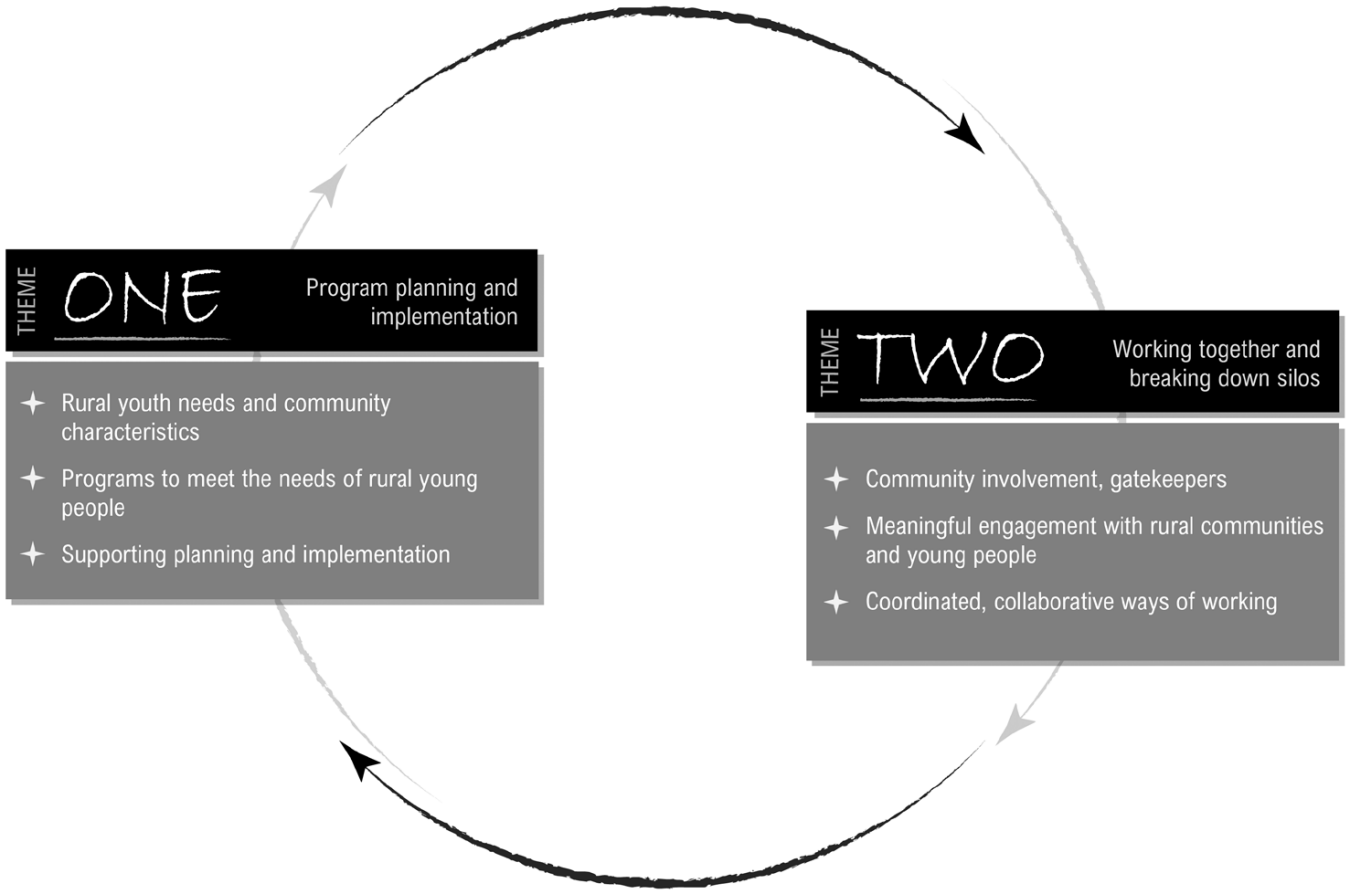
Suicide prevention for young people in rural areas: Themes and subthemes.

This study explored the role of communities in rural youth suicide prevention, focusing on two key research questions: (1) Who is best placed to implement and participate in prevention efforts? and (2) How can communities be effectively supported in these endeavours? Findings from 37 participants, aged 29–72 years (*M*=46, SD=9.6), with diverse professional roles and lived experiences of suicide (48.6%), are structured around these questions (additional demographic characteristics provided in Supporting Information 4). Two primary themes emerged: program planning and implementation, and working together to break down silos. These themes are illustrated in Figure 1, with sub‐themes explored further below in relation to the research questions.

### 3.1 Who to Implement and Participate in Prevention Efforts?

#### 3.1.1 Rural Youth Needs and Community Characteristics

Participants consistently emphasised the importance of tailoring prevention efforts to the unique characteristics of rural communities. Rural areas were described as relationship‐based, where trust, confidentiality and connection are crucial. *‘All about connection… trust…privacy and confidentiality’* (Participant 13—Research, South Australia). However, stigma surrounding mental health and suicide often deters help‐seeking behaviours. ‘*You live in a small town, and someone drives past the hospital… there's still a stigma attached to it’* (Participant 7—Suicide Prevention Program Provider, Queensland).

Findings indicate that prevention efforts need to consider cultural influences, such as intergenerational trauma in Aboriginal and Torres Strait Islander communities. ‘*Definitely for us Aboriginal and Torres Strait Islander people…intergenerational trauma that sits with those families and communities…*’ (Participant 27—Research, New South Wales). Figure 1 illustrates how social and cultural factors intersect with youth needs, highlighting the value of localised, culturally appropriate interventions. Social and cultural factors profoundly shape the needs of young people, influencing their identities, opportunities, and access to resources. Social elements such as family structure, socioeconomic status, and peer relationships interact with cultural influences like heritage, values and stigma. For example, Aboriginal and Torres Strait Islander youth and migrant youth often navigate complex dual identities, while cultural stigmas surrounding mental health can hinder access to support. The intersection of these factors creates unique challenges, particularly for marginalised groups, necessitating tailored approaches. By grounding programs in the specific contexts of the populations in focus, such initiatives leverage local knowledge, traditions, and networks to build trust and foster engagement. Such approaches not only address immediate needs but also promote resilience and equity, creating sustainable solutions for diverse youth populations, with community‐led approaches able to effectively address these needs [27, 28].

#### 3.1.2 Community Involvement and Gatekeepers

Peers play a pivotal role in supporting young people, often serving as the first point of contact when a young person reaches out. Research consistently shows that young people are more likely to confide in friends than in professionals [28], highlighting the critical need to harness the potential of peer relationships in support and referral systems. The informal nature of these interactions often provide a safe, non‐judgemental space, making it easier for young people to express their concerns. As one participant described, responding to a friend *‘compassionately, thoughtfully…’* can significantly impact their wellbeing and willingness to seek further help (Participant 13—Research, South Australia).

Given this dynamic, integrating peer‐led initiatives into broader support frameworks is a powerful strategy. Peer‐led programs leverage the unique ability of young people to relate to one another, creating an environment where individuals feel understood and valued. For example, youth‐led mental health programs often include peer counsellors or mentors who are trained to provide basic support and guidance for further, formal supports, while also fostering a sense of connection. These initiatives also help to normalise discussions about mental health, reducing stigma and encouraging help‐seeking behaviours among young people. As an example, the Live4Life rural youth mental health and suicide prevention model has a central peer component, with Crews made up of peers in year nine leading efforts within their schools and local communities [27], providing connections to further support if needed.

Incorporating the voices of those with lived experience further strengthens the impact of peer‐led approaches. Lived experience advocates bring authenticity and insight, allowing connections with young people in ways that traditional professionals may not be capable of. The involvement of people with lived experience ensures that support systems are not only relatable but also grounded in the realities of young people's experiences. By sharing their journeys, these individuals can inspire hope and resilience, while shaping interventions that are both practical and empathetic. Programs like #chatsafe illustrate the potential of co‐designed strategies to engage young people effectively [29], involving them as active partners in the planning and implementation. By valuing their insights and lived experiences, these approaches ensure interventions are relevant, responsive, and reflective of the needs of young people. The co‐design process fosters a sense of ownership and builds trust, making young people more likely to engage with and benefit from programme outcomes. For instance, youth‐led workshops or advisory panels create platforms for meaningful dialogue, addressing gaps that traditional approaches might overlook. This collaborative process not only enhances the relevance of programs but also improves their sustainability and impact, ensuring they align with the priorities of the communities they serve.

**FIGURE 2:**
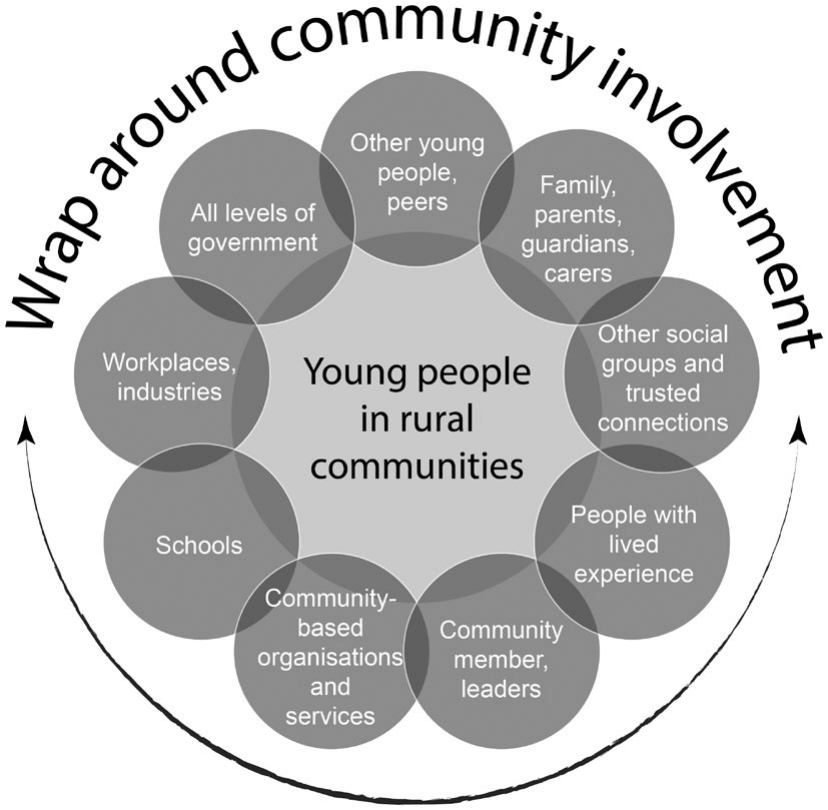
Rural communities as wraparound support for young people.

Gatekeepers such as teachers, sports coaches, and business owners were identified as central to prevention efforts. These trusted individuals often serve as informal support networks. *‘We can loosely say gatekeepers… the butcher… the newsagent… if people were trained in… it could be a very strong protective factor in a small community’* (Participant 15—Policy, Tasmania). Schools, described as community hubs, were seen as critical for providing safe spaces and early intervention. Figure 2 visualises the wraparound support offered by these community networks, demonstrating how collaboration among schools, families and gatekeepers can enhance prevention efforts.

The wraparound support provided by community networks plays a crucial role in addressing youth needs. Collaboration among schools, families, and key community gatekeepers strengthens prevention efforts by creating a cohesive support system. For example, schools can partner with families to identify early signs of distress, while gatekeepers such as local leaders or cultural elders can bridge gaps by offering culturally informed guidance. This integrated approach ensures that young people receive appropriate and comprehensive support, addressing both immediate challenges and underlying risk factors [28]. By leveraging the unique strengths of each stakeholder, such collaborations enhance the effectiveness and sustainability of prevention initiatives, building community capacity and fostering a more supportive environment for youth.

### 3.2 How Can Communities Be Effectively Supported in These Endeavours?

#### 3.2.1 Program Planning and Implementation

To effectively support communities, participants emphasised the need for adaptable, multi‐layered programs tailored to local needs. Highlighting the importance of choice, one participant noted, *‘A range of options…choice is a really important part’* (Participant 14—Service and Suicide Prevention Programme Provider, Tasmania). Such programmes recognise the diversity of communities, offering flexible pathways that align with specific circumstances and preferences.

Multi‐layered approaches combine immediate interventions with long‐term support, such as crisis services paired with education and ongoing counselling. Tailoring these programmes to local contexts enhances accessibility and relevance, particularly in culturally or geographically distinct areas. By prioritising flexibility and inclusivity, these programmes empower communities to engage meaningfully, fostering resilience and sustainable well‐being.

Programs addressing stigma must use culturally appropriate language to resonate with young people and their communities. Stigma around topics like suicide can be particularly pronounced among young people, where peer and cultural pressures often amplify fears of judgement or rejection. As one participant highlighted, *‘In some communities…there still is quite a high degree of stigma attached to suicide… it's important to talk about appropriate language and de‐stigmatising it*’ (Participant 13—Research, South Australia). Using language that is inclusive and sensitive helps young people feel seen and understood, reducing barriers to seeking help.

For young people, culturally tailored messaging is crucial to fostering open discussions within their communities. By recognising the unique challenges youth face—such as balancing familial, social and cultural expectations with modern influences—programmes can create safe, supportive environments for dialogue. De‐stigmatising approaches empower young people to speak out, support their peers, and engage with resources, building trust and resilience within their communities.

Protective measures, such as mindfulness and resilience programs, are vital for promoting the well‐being of young people. These initiatives equip youth with practical tools to manage stress, regulate emotions and navigate challenges. As one participant noted, *‘Mindfulness and even yoga in schools, resilience programs… mental wellbeing and emotional coaching’* (Participant 18—Service Provider, Victoria), these approaches support both mental and emotional development. Embedding mindfulness and resilience programmes in schools ensures that young people can access these resources in familiar and supportive environments. Activities like yoga, mindfulness exercises, and emotional coaching help youth build self‐awareness, self‐care and coping skills, fostering long‐term resilience. By integrating such measures, schools and communities can play an active role in cultivating mental well‐being and equipping young people with lifelong strategies for managing adversity.

Safety planning and postvention efforts are also critical components of supporting young people and their communities. These measures not only provide immediate support following a crisis but also help prevent further incidents by addressing underlying risks. As one participant observed, *‘Copycat, clusters… it is postvention… it's actually prevention as well’* (Participant 12—Service Provider, Tasmania), underscoring the dual role postvention plays in both recovery and prevention. These finding closely align with the National Suicide Prevention Strategy's [10] focus on integrated community responses, ensuring a coordinated approach to reducing harm. By involving families, schools, and local organisations, safety planning and postvention create a network of care that fosters healing while mitigating the risk of contagion or clusters. Such proactive, community‐centred strategies are essential for promoting resilience and reducing the long‐term impact of suicide in young people's lives.

#### 3.2.2 Supporting Capacity‐Building and Sustainability

Participants emphasised the importance of equipping communities with resources and training to better support young people. Administrative burdens and burnout were identified as significant challenges to sustaining these efforts. As one participant explained, *‘When the community members are bogged down with strategic planning… there's no action… Burnout is a barrier*’ (Participant 12—Service Provider, Tasmania). These barriers can directly impact the availability of programmes for young people, limiting their access to essential support.

Embedding programmes into daily community activities was proposed as a way to enhance sustainability and relevance for young people. For example, integrating mental health and resilience‐building initiatives into school curriculums or local youth organisations can ensure these programmes are both accessible and part of routine interactions. As one participant noted, *‘It has to be part of daily service… part of your business’* (Participant 17—Research, Northern Territory). This approach not only reduces the strain on community members but also creates consistent opportunities for young people to engage with support systems, fostering stronger connections and long‐term well‐being.

Lived experience insights were highlighted as essential for improving outcomes for young people and their communities. These perspectives provide authentic and relatable contributions that enhance the relevance and impact of programmes. As one participant explained, *‘Lived experience… was a significant part of that impact… helping to save people's lives down the track…’* (Participant 13—Research, South Australia). By integrating these insights, programmes can better address the specific needs of young people, fostering trust and creating more meaningful interventions while building local community capacity.

Continuous evaluation was also recognised as crucial for refining and improving programme effectiveness. Regular assessment ensures that initiatives remain responsive to the changing needs of young people and their communities. By identifying gaps and measuring outcomes, evaluation helps to adapt and enhance programmes over time, ensuring they deliver sustained and meaningful impact. Together, lived experience and evaluation create a dynamic framework for ongoing improvement and sustainability.

#### 3.2.3 Breaking Down Silos and Fostering Collaboration

Breaking down silos was identified as crucial for maximising resources and providing holistic support to young people and their communities. Silos between services often lead to fragmented efforts, limiting the effectiveness of interventions. As one participant noted, *‘A coordinated effort to make sure that we can wrap around, individuals, communities, and families that need it’* (Participant 16—Policy/Research, Queensland), emphasising the importance of collaboration in delivering comprehensive care.

Figure 2 illustrates how coordination among schools, health services and community organisations can unify fragmented services. This collaborative approach addresses resource gaps by pooling expertise and fostering shared accountability. By working together, these stakeholders can create integrated support systems that provide young people and their families with seamless access to the resources and care they need, ensuring more effective and sustainable outcomes.

Partnerships with cultural leaders, elders, and young people were emphasised as essential for addressing the needs of youth in rural and Aboriginal communities. These partnerships help ensure that responses are culturally appropriate and rooted in community values. As one participant highlighted, ‘*In Aboriginal communities… connection with those Elders… to ensure the response… is collaborative…’* (Participant 1—Service and Suicide Prevention Programme Provider, Western Australia). Engaging elders and cultural leaders creates a bridge between traditional knowledge and modern approaches, fostering relevance and respect within the community. Genuine co‐design processes involving young people further enhance programme success by fostering ownership and trust [30]. When youth are actively included in shaping initiatives, they are more likely to engage with and champion them. This approach builds a sense of connection, empowerment and ensures programmes address the unique challenges faced by rural and Aboriginal communities [31]. By uniting cultural wisdom, community collaboration, and youth leadership, these partnerships create sustainable and impactful support systems tailored to the needs of young people in rural communities.

### 3.3 Summary

In summary, this study highlights the pivotal role of trusted community members and local organisations in rural youth suicide prevention. The research journey revealed the profound impact community‐led, culturally responsive approaches have in addressing the unique challenges faced by rural youth. The reliance on gatekeepers, peers, and schools underscores the importance of grassroots involvement, as these trusted figures are integral to fostering early intervention and creating supportive environments.

Findings also emphasised the complexity of rural suicide prevention, requiring a holistic approach that accounts for intergenerational trauma, stigma, and cultural diversity. This reflection affirms the need for systemic collaboration, breaking down silos to build cohesive networks of support. The process of engaging with participants revealed their commitment to addressing these challenges, providing valuable insights into how communities can harness their strengths to sustain prevention efforts. The use of lived experience voices throughout the research offered a rich perspective, reinforcing the necessity of genuine co‐design in programme development. Ultimately, the study reflects the resilience and adaptability of rural communities, highlighting their potential to lead transformative change in youth suicide prevention.

### 3.4 Implications for Policy and Practice

Findings support the literature and underscore the importance of community‐led, systems‐based approaches to suicide prevention [13, 15],particularly tailored for young people [28]. Existing initiatives offer valuable insights and frameworks for integrating local knowledge and lived experience into policy and practice,currently being trialled in rural communities and showing promise [32]. Schools as community hubs, and workplaces as duty‐of‐care leaders, are central to early intervention efforts. Aligning these approaches with broader determinants of health, including stigma reduction and enhancing service accessibility, is critical for systemic change. Policies should prioritise funding for local partnerships, capacity‐building initiatives, and integrated service networks, including communities of practice and building on existing programmes and strategies [9, 15]. Existing national strategies and standards, including Suicide Prevention Australia's quality improvement guidelines [33] as well as the development of *Best Practice Guideline for Youth Suicide Prevention in Rural Australian Communities*, currently being developed by the lead author, provide actionable frameworks for enhancing rural youth suicide prevention efforts.

#### 3.4.1 Study Limitations

Variable participant numbers across the state, demographic, and participant groups mean participant views may not be generalisable to the whole population of people working in the field or across all communities or rural areas. In addition, participants represent the views of only professionals working in the field, aged 29 and over, with experience in programme planning and implementation working with young people, rather than the views of young people themselves, highlighting this area still in need of research focus.

## 4 Conclusion

Across rural Australian communities, suicide prevention programmes for young people need the involvement of communities to lead efforts and provide the wrap‐around support needed. These insights need to inform policy development and programmes, recognising that the journey of young people is impacted by a complex interplay of rural, personal, and social factors, and programme efforts need to reflect these. Programmes and support processes rural communities need to plan and implement prevention efforts therefore need to be flexible and inclusive building on the strengths of communities to ensure the best use of what resources are scarcely available.

## Author Contributions

**Laura Grattidge:** conceptualisation, investigation, funding acquisition, writing – original draft, writing – review and editing, visualisation, validation, methodology, software, formal analysis, project administration, data curation, resources. **Ha Hoang:** supervision, resources, formal analysis, writing – review and editing, validation, methodology, funding acquisition, conceptualisation. **Jonathan Mond:** conceptualization, writing – review and editing, supervision, funding acquisition. **Denis Visentin:** writing – review and editing, supervision. **David Lees:** writing – review and editing, conceptualisation. **Stuart Auckland:** writing – review and editing, funding acquisition.

## Ethics Statement

Ethics approval was granted by the University of Tasmania Human Research Ethics Committee (Project ID 23582).

## Conflicts of Interest

The authors declare no conflicts of interest.

## Supporting information


Supporting Information 1.

Supporting Information 2.

Supporting Information 3.

Supporting Information 4.

## Data Availability

The data that support the findings of this study are available on request from the corresponding author. The data are not publicly available due to privacy or ethical restrictions.
