# Supplementary material for: The Community's Role in Rural Youth Suicide Prevention: Perspectives From the Field
Source: Aust J Rural Health. 2025 Mar 10;33(2):e70024. doi: 10.1111/ajr.70024 (PMC11891953; doi:10.1111/ajr.70024)
Supplement: Supplementary file 2 — Supporting Information 2. [file AJR-33-0-s001.pdf]

Supplementary file 2.

Table 1: Consolidated criteria for reporting qualitative studies (COREQ): 32-item checklist

**No**	**Item**	**Guide questions/description**	**Author response**
**Domain 1: Research team and reflexivity**			
**Personal Characteristics**			
1.	Interviewer/facilitator	Which author/s conducted the interview or focus group?	Author 1
2.	Credentials	What were the researcher's credentials? *E.g. PhD, MD*	Author 1 - MPH, BA/BCom, Bphil
3.	Occupation	What was their occupation at the time of the study?	Project Manager; PhD Candidate
4.	Gender	Was the researcher male or female?	Female
5.	Experience and training	What experience or training did the researcher have?	Researchers who conducted interviews and focus groups have extensive experience undertaking qualitative research and interviews and focus groups with vulnerable population groups
**Relationship with participants**			
6.	Relationship established	Was a relationship established prior to study commencement?	There was no relationship between the participants and researcher prior to the study
7.	Participant knowledge of the interviewer	What did the participants know about the researcher? e.g. *personal goals, reasons for doing the research*	Through reading the Participant Information Statement and as confirmed at the beginning of the interview or focus group, the participants were aware of the purpose of the study
8.	Interviewer characteristics	What characteristics were reported about the interviewer/facilitator? e.g. *Bias, assumptions, reasons and interests in the research topic*	No conflicts of interest were disclosed by any of the authors
**Domain 2: study design**			
**Theoretical framework**			
9.	Methodological orientation and Theory	What methodological orientation was stated to underpin the study? *e.g. grounded theory, discourse analysis, ethnography, phenomenology, content analysis*	The study used inductive thematic analysis
**Participant selection**			
10.	Sampling	How were participants selected? *e.g. purposive, convenience, consecutive, snowball*	Purposive and snowball
11.	Method of approach	How were participants approached? e.*g. face-to-face, telephone, mail, email*	Participants were approached through email
12.	Sample size	How many participants were in the study?	37
13.	Non-participation	How many people refused to participate or dropped out? Reasons?	Two participants dropped out
**Setting**			
14.	Setting of data collection	Where was the data collected? e.*g. home, clinic, workplace*	Interviews and focus groups run through zoom. Participants were located in a range of settings including homes, workplaces, public spaces. Face-to-face interview conducted at participants workplace.
15.	Presence of non-participants	Was anyone else present besides the participants and researchers?	There was no one else present at the interviews and focus groups besides the participants and researchers

16.	Description of sample	What are the important characteristics of the sample? *e.g. demographic data, date*	All participants had experience working in this field, either service provider or suicide prevention program provider, research or policy role. A total of 37 people participated in 32 individual interviews and two focus groups (ages 29-72 (SD=9.59); female 62.2%; lived experience 48.6%).
**Data collection**			
17.	Interview guide	Were questions, prompts, guides provided by the authors? Was it pilot tested?	A topic guide was utilised by researchers and the questions were pilot tested
18.	Repeat interviews	Were repeat interviews carried out? If yes, how many?	No repeat interviews were carried out
19.	Audio/visual recording	Did the research use audio or visual recording to collect the data?	Interviews and focus groups were audio recorded
20.	Field notes	Were field notes made during and/or after the interview or focus group?	Field notes were taken by the research team at interviews and focus groups
21.	Duration	What was the duration of the interviews or focus group?	On average, interviews and focus groups were 51 minutes
22.	Data saturation	Was data saturation discussed?	Data saturation was discussed by the research team in regular team meetings and regularly by those collecting and analysing data.
23.	Transcripts returned	Were transcripts returned to participants for comment and/or correction?	Given the sensitive nature of the topic and confidentiality issues with small groups, transcripts were not returned to participants for comment unless requested. No focus group or interview participants requested data.
**Domain 3: analysis and findings**			
**Data analysis**			
24.	Number of data coders	How many data coders coded the data?	Data were coded by one researcher and confirmed by a second.
25.	Description of the coding tree	Did authors provide a description of the coding tree?	Authors described all themes identified from the data (Figure 1)
26.	Derivation of themes	Were themes identified in advance or derived from the data?	Themes were derived from the data
27.	Software	What software, if applicable, was used to manage the data?	NVivo software was used to analyse data
28.	Participant checking	Did participants provide feedback on the findings?	Participants provided feedback on the findings at presentations made by the researcher, for example the Australian Public Health Conference 2023
**Reporting**			
29.	Quotations presented	Were participant quotations presented to illustrate the themes / findings? Was each quotation identified? e.*g. participant number*	Each quote was identified with participant number, jurisdiction and group representation
30.	Data and findings consistent	Was there consistency between the data presented and the findings?	Views and findings were consistent across participant groups. There was consistency between the data presented and the findings.

31.	Clarity of major themes	Were major themes clearly presented in the findings?	Two major themes are clearly presented in the findings: 1) Program planning and implementation, and 2) Working together and breaking down silos
32.	Clarity of minor themes	Is there a description of diverse cases or discussion of minor themes?	Minor themes are discussed within the major themes presented throughout the manuscript
