# Supplementary material for: The Community's Role in Rural Youth Suicide Prevention: Perspectives From the Field
Source: Aust J Rural Health. 2025 Mar 10;33(2):e70024. doi: 10.1111/ajr.70024 (PMC11891953; doi:10.1111/ajr.70024)
Supplement: Supplementary file 3 — Supporting Information 3. [file AJR-33-0-s003.pdf]

ABORIGINAL AND TORRES STRAIT ISLANDER QUALITY APPRAISAL TOOL

ABORIGINAL AND TORRES STRAIT ISLANDER QUALITY APPRAISAL TOOL

Answer either "Yes", "Partially", "No" or "Unclear" to each question

Article citation: The community's role in rural youth suicide prevention: perspectives from the field

Date: 17/2/2024

Reviewer's name: ____________________

Question	Yes	Partially	No	Unclear
1. Did the research respond to a need or priority determined by the community?	✔			
2. Was community consultation and engagement appropriately inclusive?		✔		
3. Did the research have Aboriginal and Torres Strait Islander research leadership?			✔	
4. Did the research have Aboriginal and Torres Strait Islander governance?			✔	
5. Were local community protocols respected and followed?				✔
6. Did the researchers negotiate agreements in regards to rights of access to Aboriginal and Torres Strait Islander peoples' existing intellectual and cultural property?			✔	
7. Did the researchers negotiate agreements to protect Aboriginal and Torres Strait Islander peoples' ownership of intellectual and cultural property created through the research?			✔	
8. Did Aboriginal and Torres Strait Islander peoples and communities have control over the collection and management of research materials?			✔	
9. Was the research guided by an Indigenous research paradigm?			✔	
10. Does the research take a strengths-based approach, acknowledging and moving beyond practices that have harmed Aboriginal and Torres Strait peoples in the past?	✔			
11. Did the researchers plan and translate the findings into sustainable changes in policy and/or practice?	✔			
12. Did the research benefit the participants and Aboriginal and Torres Strait Islander communities?				✔
13. Did the research demonstrate capacity strengthening for Aboriginal and Torres Strait Islander individuals?		✔		
14. Did everyone involved in the research have opportunities to learn from each other?		✔		
