# Supplementary material for: The Community's Role in Rural Youth Suicide Prevention: Perspectives From the Field
Source: Aust J Rural Health. 2025 Mar 10;33(2):e70024. doi: 10.1111/ajr.70024 (PMC11891953; doi:10.1111/ajr.70024)
Supplement: Supplementary file 4 — Supporting Information 4. [file AJR-33-0-s004.pdf]

Supplementary file 4

Table S4.1. Participant demographic characteristics.

**Age** * (*n* = 35), range = 29–72, ***MEAN*** = 46 (*SD* = 9.6)		
**Gender**	***N***	***%***
Female	23	*62.2*
Male	11	*29.7*
Other (non-binary)	3	*8.1*
**State**		
South Australia	1	*2.7*
Northern territory	2	*5.4*
Victoria	4	*10.8*
Western Australia	6	*16.2*
New South Wales	7	*18.9*
Queensland	8	*21.6*
Tasmania	9	*24.3*
**Lived experience**		
Yes	20	*48.6*
No	9	*24.3*
Prefer not to say	6	*16.2*
Non response	2	*5.4*
**Aboriginal and/or Torres Strait Islander**		
Aboriginal	2	*5.6*
Neither Aboriginal nor Torres Strait Islander	34	*91.9*
Non response	1	*2.7*
**Role in community-based suicide prevention**		
Community-level service provider	18	*48.6*
Suicide prevention program provider	7	*18.9*
Policy or research	12	*32.5*

Abbreviations: *n*, number; *SD*, Standard deviation; * Missing data for age for two participants.
